# Evaluating the prognostic value of tumor deposits in non-metastatic lymph node-positive colon adenocarcinoma using Cox regression and machine learning

**DOI:** 10.1007/s00384-024-04671-2

**Published:** 2024-06-26

**Authors:** Zhen Zheng, Hui Luo, Ke Deng, Qun Li, Quan Xu, Kaitai Liu

**Affiliations:** 1 Department of Chemoradiation Oncology, The Affiliated Lihuili Hospital of Ningbo University, 57 Xingning RoadZhejiang Province, Ningbo, China; 2 Department of Colorectal Surgery, The Affiliated Lihuili Hospital of Ningbo University, Zhejiang Province, Ningbo, China; 3 Department of Otolaryngology Head and Neck Surgery, The Affiliated Lihuili Hospital of Ningbo University, Zhejiang Province, Ningbo, China

**Keywords:** Adenocarcinoma of colon, Machine learning, Nomogram, Cox regression, Tumor deposit, Prognosis

## Abstract

**Background:**

The 8th AJCC TNM staging for non-metastatic lymph node-positive colon adenocarcinoma patients(NMLP-CA) stages solely by lymph node status, irrespective of the positivity of tumor deposits (TD). This study uses machine learning and Cox regression to predict the prognostic value of tumor deposits in NMLP-CA.

**Methods:**

Patient data from the SEER registry (2010–2019) was used to develop CSS nomograms based on prognostic factors identified via multivariate Cox regression. Model performance was evaluated by c-index, dynamic calibration, and Schmid score. Shapley additive explanations (SHAP) were used to explain the selected models.

**Results:**

The study included 16,548 NMLP-CA patients, randomized 7:3 into training (*n* = 11,584) and test (*n* = 4964) sets. Multivariate Cox analysis identified TD, age, marital status, primary site, grade, pT stage, and pN stage as prognostic for cancer-specific survival (CSS). In the test set, the gradient boosting machine (GBM) model achieved the best C-index (0.733) for CSS prediction, while the Cox model and GAMBoost model optimized dynamic calibration(6.473) and Schmid score (0.285), respectively. TD ranked among the top 3 most important features in the models, with increasing predictive significance over time.

**Conclusions:**

Positive tumor deposit status confers worse prognosis in NMLP-CA patients. Tumor deposits may confer higher TNM staging. Furthermore, TD could play a more significant role in the staging system.

## Instruction

Colorectal cancer (CRC) remains a major public health burden, ranking 3rd in global cancer incidence and 2nd in mortality with over 1 million new cases and 550,000 deaths annually [1, 2]. With improving diagnosis and treatment, CRC survival has increased, underscoring the need for accurate prognostic awareness to inform therapeutic decision-making. The American Joint Committee on Cancer (AJCC) TNM staging system is the standard for guiding CRC treatment and prognosis [3]. However, for non-metastatic lymph node-positive colon adenocarcinoma (NMLP-CA), the current AJCC guidelines do not fully capture the prognostic impact of tumor deposits (TD) [4]. Emerging evidence indicates patients with both TD and lymph node metastases (LNM) have poorer outcomes compared to either condition alone [5, 6]. While some studies explore TD prognostic value in stage III colon cancer [7, 8], this population includes N1c patients, potentially introducing bias. The prognostic role of TD specifically in NMLP-CA remains unclear.

Moreover, current research on the prognosis assessment of TD primarily relies on classical statistical models, such as Cox regression. Cox models require adherence to the proportional hazards assumption, which means that the overall quality of these models may not have reached the optimal state in some cases. Machine learning is a novel form of artificial intelligence that has found extensive application in medical data analysis, making it a potent tool for enhancing clinical strategies [9–11]. In numerous studies where the dependent variable was categorical, machine learning has demonstrated superior prediction performance compared to traditional models [10, 12–14]. *However, few studies compare survival prediction models to assess TD in NMLP-CA, where outputs include survival status and time. It remains unclear if new machine learning models outperform Cox regression for this purpose*[6, 15–17]*.* Our aim is to assess the impact of TD on the prognosis of NMLP-CA and to compare traditional prognostic models with machine learning models to identify an optimal prognostic model for NMLP-CA.

## Materials and methods

### Patients and data sources

**Fig. 1:**
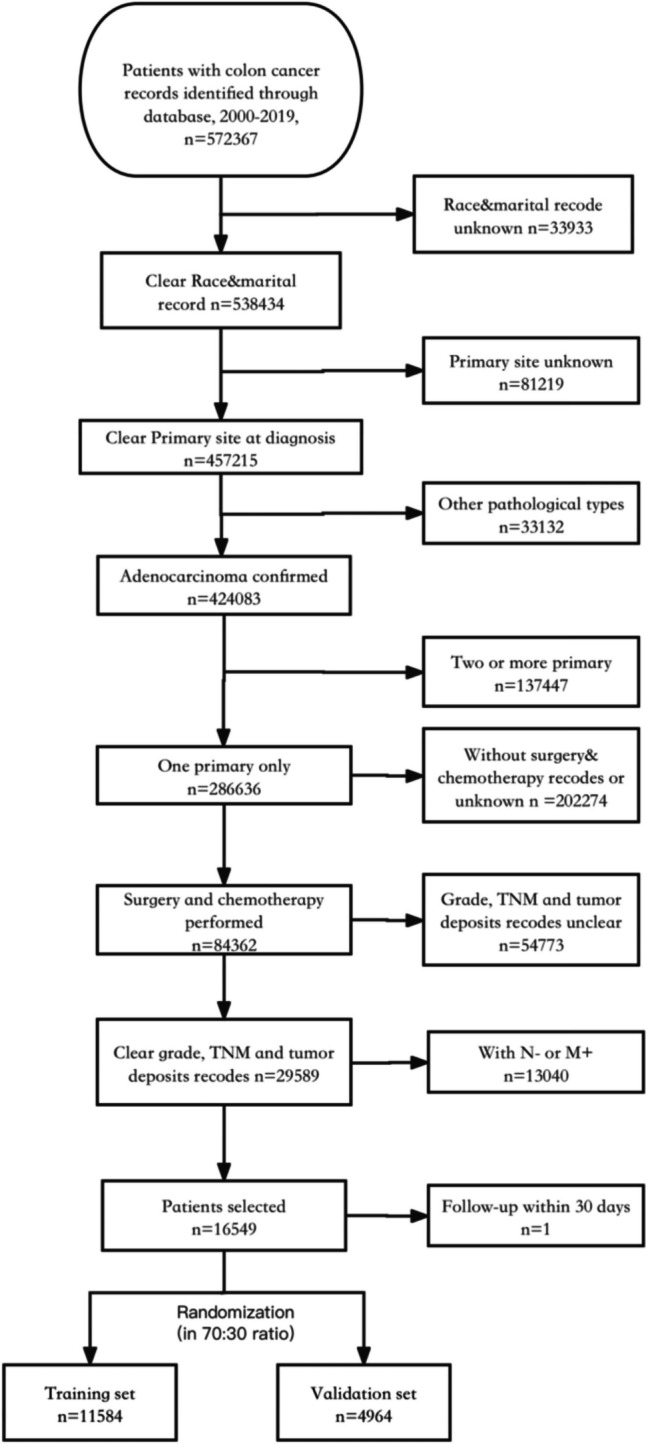
The inclusion criteria flowchart of recruited patients in SEER database

This study analyzed non-metastatic lymph node-positive colon cancer patients using data from the Surveillance, Epidemiology, and End Results (SEER) registry from 2010–2019. According to the AJCC/UICC TNM7 and TNM8 staging systems, TDs are defined as cancerous nodules located in the lymph drainage area of the peritumoral fatty tissue, characterized by the histologically proven absence of residual lymphatic tissue and regional lymph node metastasis [18–20]. The defining features of TDs include their proximity to the main tumoral front, size, and the absence of a lymphoid rim and fibrous capsule, rather than the presence of lymphoid structures or lymph nodes within the surrounding adipose tissue [21]. Inclusion criteria were as follows: (1) colon cancer as the sole primary tumor with pathologically confirmed adenocarcinoma; (2) availability of demographic variables, including age, sex, marital status, and race; (3) access to clinical pathological information, encompassing tumor site, TNM staging, histological grade, TD, and specific treatment details; (4) acquisition of survival time and survival status. The exclusion criteria included the following: (1) patients with other primary malignant tumors; (2) patients who did not undergo surgery and chemotherapy; (3) patients with distant metastasis or regional lymph node negativity; (4) individuals with incomplete follow-up information; (5) patients with less than 1-month postoperative survival, in order to minimize confounding from surgical mortality. In this study, The primary outcome was cancer-specific survival (CSS), measured from diagnosis to cancer-related death. Figure 1 illustrates the data selection process. In accordance with the Equator Network guidelines, we followed the Strengthening the Reporting of Observational Studies in Epidemiology (STROBE) guidelines for this study.

### Establishment of predictive model

This study performed a comparative analysis of clinicopathologic characteristics from the SEER database and assessed prognostic factors via univariate and multivariate logistic regression. Six machine learning models were developed, including Cforest, gradient boosting machine (GBM), generalized linear model boosting (GLMboost), random forestSRC (Rfsrc), generalized additive model boosting (GAMboost), and deep learning survival analysis (DeepSurv). The data was split 70:30 into training and test sets. Ten-fold cross-validation tuned hyperparameters during training to ensure model stability. The best parameters for each model in the training cohort were determined by the grid search method. Model evaluation on the test set employed C-index, dynamic calibration, and Schmid score. Comparative assessment identified optimal techniques for predicting prognosis in this cohort.

### Statistical analysis

Data extraction from SEER utilized the SEER*Stat software (version 8.4.2). Analysis was performed in R (version 4.3.1). Continuous variables were analyzed by Wilcoxon rank-sum test, while categorical variables used chi-squared or Fisher’s exact test. Univariate and multivariate logistic regression assessed the prognostic value of TDs at a significance level of *p* < 0.05. Adjusted odds ratios (OR) and 95% confidence intervals (CI) were computed.

The modeling process was implemented using the mlr3 package (version 0.16.1) in R. We tested the training set with Cox, Cforest, GBM, GLMboost, Rfsrc, GAMboost, and Deepsurv to establish prediction models. Additionally, Shapley additive explanations (SHAP) were utilized to interpret these models [22, 23]. SHAP is a model interpretation package developed in R that can explain the output of any machine-learning model. Permutation importance was calculated using the survex package (version 1.1.3) in R.

## Results

### Baseline characteristics

This study analyzed 572,367 colon cancer patients from the SEER database between 2010 and 2019. After applying exclusion criteria, 16,548 patients remained. Figure 1 displays the data screening flowchart. The baseline characteristics of the patients are summarized in Table 1. After screening, the median follow-up time for patients was 51 months (range 1–119). The data was randomized 7:3 into training (*n* = 11,584) and testing (*n* = 4964) sets. No significant differences or survival discrepancies (*p* = 0.96) existed between the sets.

**Table 1:** Baseline demographic and clinical characteristics of colon cancer patients with postoperative chemotherapy

	Total *N* = 16,548	Training set *N* = 11,584	Testing set *N* = 4964	*p*-value
Age (*n*, %)				0.95
≤60	7676 (46.4%)	5336 (46.1%)	2340 (47.1%)	
>60	8872 (53.6%)	6248 (53.9%)	2624 (52.9%)	
Race (*n*, %)				0.62
White	1250 (75.6%)	8739 (75.4%)	3769 (75.9%)	
Other	4040 (24.4%)	2845 (24.6%)	1195 (24.1%)	
Marital status (*n*, %)				0.15
Married	1001 (60.5%)	7039 (60.8%)	1987 (40.0%)	
Other	6532 (39.5%)	4545 (39.2%)	2977 (60.0%)	
Primary site (*n*, %)				0.59
Right	9257 (55.9%)	6518 (56.3%)	2739 (58.4%)	
Left	7291 (44.1%)	5066 (43.7%)	2225 (41.6%)	
Grade (*n*, %)				0.55
I	868 (5.2%)	602 (5.2%)	266 (5.4%)	
II	11,613 (70.2%)	8181 (70.6%)	3432 (69.1%)	
III	3332 (20.1%)	2293 (19.8%)	1039 (20.9%)	
IV	735 (4.5%)	508 (4.4%)	227 (4.6%)	
T (*n*, %)				0.10
0 + 1	784 (4.8%)	552 (4.8%)	232 (4.7%)	
2	1449 (8.8%)	1000 (8.6%)	449 (9.0%)	
3	1057 (63.9%)	7439 (64.2%)	3135 (63.2%)	
4	3741 (22.5%)	2593 (22.4%)	1148 (23.1%)	
N (*n*, %)				0.26
1	1057 (63.9%)	7438 (64.2%)	3141 (63.3%)	
2	5969 (36.1%)	4146 (35.8%)	1823 (36.7%)	
Sex (*n*, %)				0.97
Female	8225 (49.7%)	5785 (49.9%)	2440 (45.1%)	
Male	8323 (50.3%)	5799 (50.1%)	2524 (54.9%)	
Tumor deposit (*n*, %)				0.67
Positive	1325 (80.1%)	9328 (80.5%)	3922 (79.0%)	
Negative	2397 (19.9%)	2256 (19.5%)	2977 (21.0%)	

#### Univariable and multivariable Cox regression analysis

**Fig. 2:**
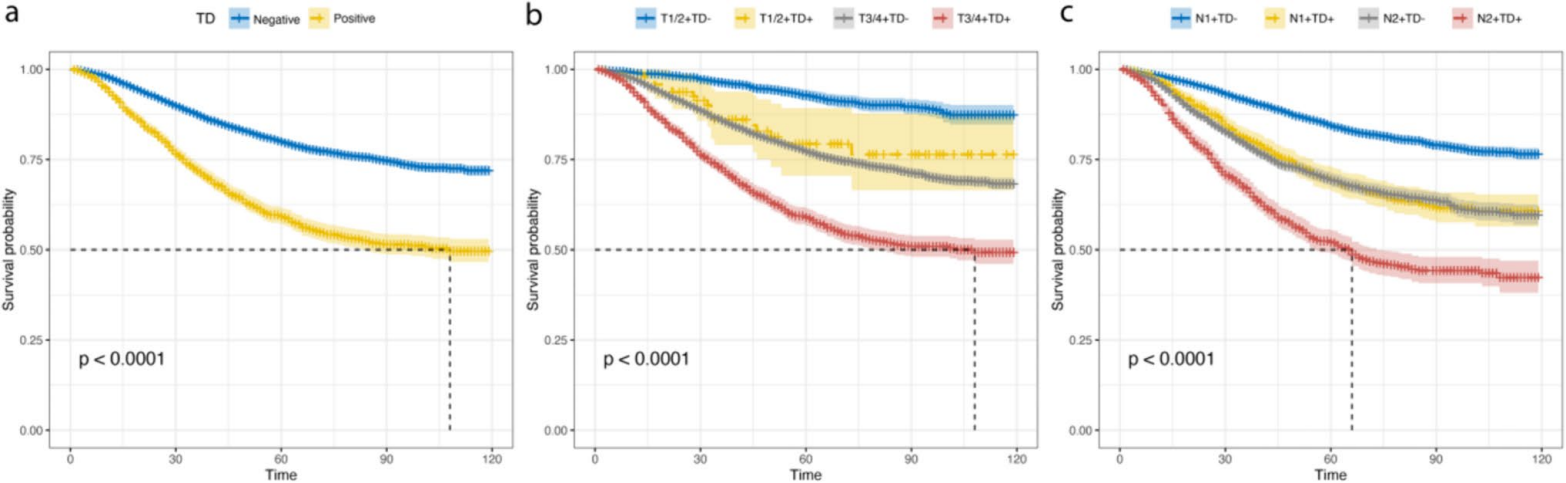
Kaplan–Meier cancer-specific survival (CSS) estimates and 95% confidence intervals for patients in groups combined with or without tumor deposit (TD) (**a**), groups with different T stages combined with or without TD (**b**), and groups with different N stages combined with or without TD in the training cohort (**c**)

**Fig. 3:**
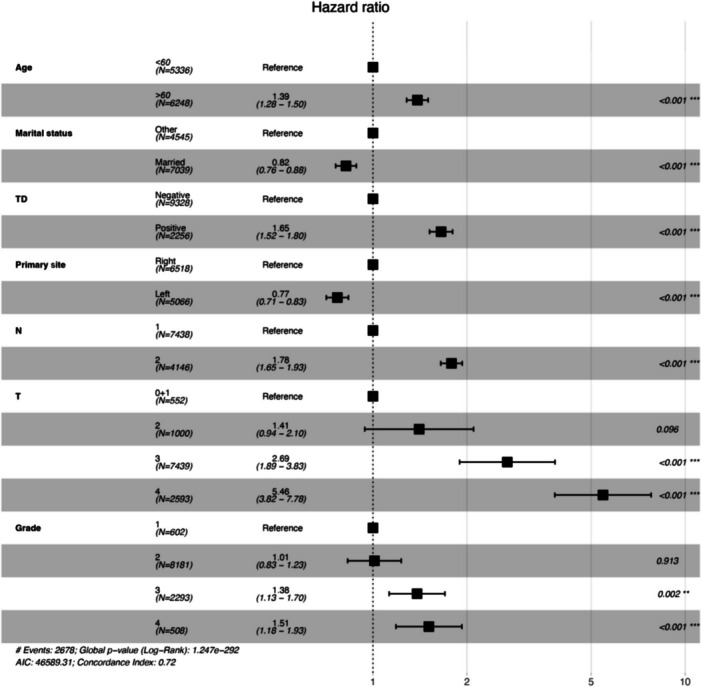
Cox proportional hazard ratios with 95% confidence intervals. There is a dashed line for an equivalent hazard ratio (HR = 1; **p < 0.01; ***p < 0.001; TD, tumor deposit)

**Fig. 4:**
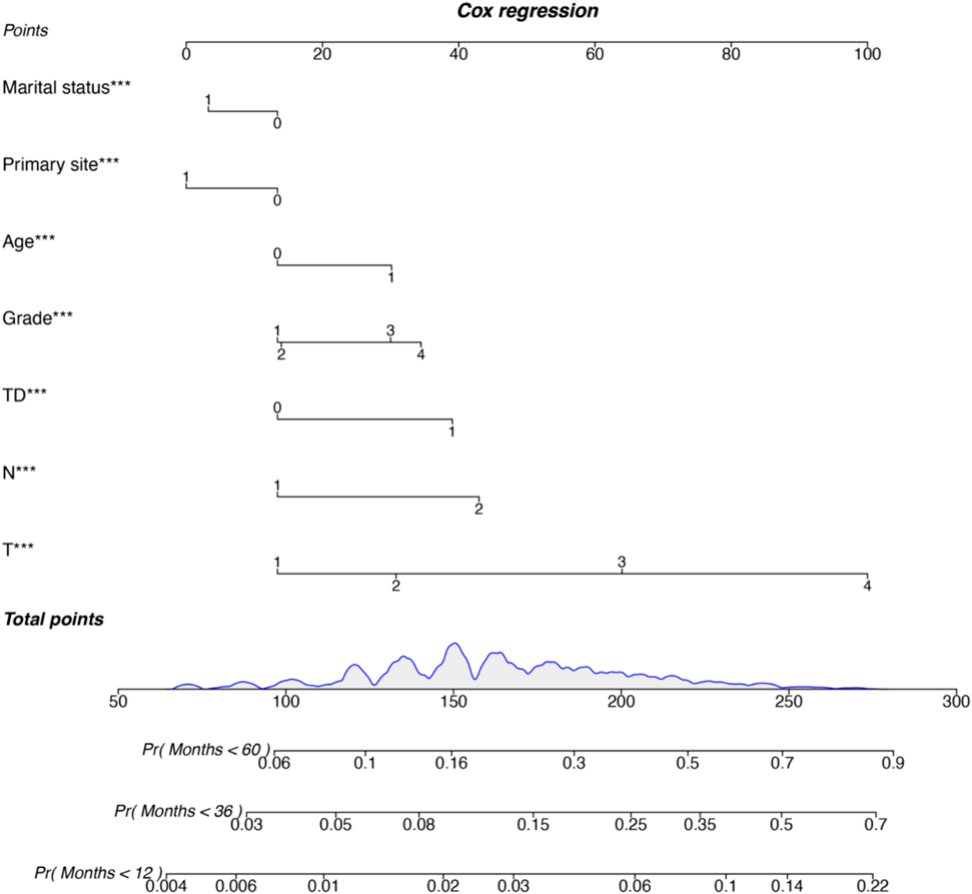
Establishment of cancer-specific survival (CSS) nomograms (**p < 0.01; ***, *p* < 0.001; TD, tumor deposit)

Univariate analysis identified age, race, marital status, primary site, TD, AJCC pT/N stage, grade, and sex as variables significantly influencing CSS (Table 2). Kaplan–Meier analysis demonstrated worse survival for patients with positive versus negative tumor deposits (*p* < 0.001) (Fig. 2a). Subgroup analyses revealed TD may respectively impact survival across T and N stages. Survival for T1 + 2 TD positive patients resembled T3 + 4 TD negative patients, while N1 TD + patients resembled N2 TD patients (Fig. 2b and c). These findings suggest TD may confer higher TNM staging and portend worse prognosis, despite not being incorporated in the current 8th edition AJCC guidelines. Multivariable Cox regression confirmed positive TD as an independent predictor of worse CSS, alongside age > 60 years, unmarried status, right-sided tumors, advanced T/N stage, and poor Grade (Fig. 3). A prognostic nomogram for 6-, 12-, and 24-month CSS was constructed using the Cox model (Fig. 4). Model discrimination assessed by the C-index was 0.717.

**Table 2:** Univariate and multivariate analysis of overall survival in the training cohort

	N	Univariate	Multivariate	
		p	HR(95% CI)	p
Age		<.001		
≤60	5336(46.1%)		Reference	
>60	6248(53.9%)		1.35(1.25-1.47)	<.001
Ethnicity		0.8		
White	8739(75.4%)			
Other	2845(24.6%)			
Marital status		<.001		
Married	7039(60.8%)		Reference	
Other	4545(39.2%)		0.77(0.71-0.83)	<.001
Primary Site		<.001		
Right	6518(56.3%)		Reference	
Left	5066(43.7%)		0.76(0.70-0.82)	<.001
Grade		<.001		
I	602(5.2%)		Reference	
II	8181(70.6%)		1.07(0.94-1.30)	0.53
III	2293(19.8%)		1.40(1.13-1.72)	<.001
IV	508(4.4%)		1.57(1.23-2.00)	<.001
T (n, %)		<.001		
0+1	552(4.8%)		Reference	
2	1000(8.6%)		1.33(0.90-1.97)	0.16
3	7439(64.2%)		2.42(1.71-3.42)	<.001
4	2593(22.4%)		4.76(3.36-6.76)	<.001
N		<.001		
1	7438(64.2%)			
2	4146(35.8%)		1.88(1.74-2.04)	<.001
Sex		0.5		
Female	5785(49.9%)			
Male	5799(50.1%)			
Tumor deposit		<.001		
Negative	9328(80.5%)		Reference	
Positive	2256(19.5%)		1.73(1.59-1.88)	<.001

*HR* hazard ratio, *CI* confidence interval

### Establishing and evaluating predictive models for estimating the prognosis

Six machine learning models (cforest, GBN, GLMboost, rfsrc, GAMboost, Deepsurv) were developed to predict CSS. The data was split 7:3 into training and testing sets. Ten-fold cross-validation tuned hyperparameters and evaluated model performance on the training data. C-index (cindex), dynamic calibration (dcalib), and Schmid score (Schmid) comparisons on the test set informed the final model selection (Table 3). GBM achieved the best cindex (0.733), Cox had optimal dynamic calibration (6.473), and GAMBoost maximized the overall performance of the Schmid score (0.285). Thus, GBM, Cox, and GAMBoost were chosen for further analysis.

**Table 3:** Model evaluation indexs in the training and test cohort for the Cox, Cforest, GBM, GLMboost, Rfsrc, GAMboost, and Deepsurv models

Models	Training cohort	Testing cohort		
	C-index	C-index	Dcalib	Schmid
COX	0.717	0.730	6.473	0.264
Cforest	0.702	0.718	8.640	0.252
GBM	0.714	0.733	2006.504	0.212
GLMboost	0.699	0.722	12.200	0.279
Rfsrc	0.712	0.727	12.155	0.277
GAMboost	0.710	0.715	13.728	0.285
Deepsurv	0.714	0.723	8.640	0.252

**Fig. 5:**
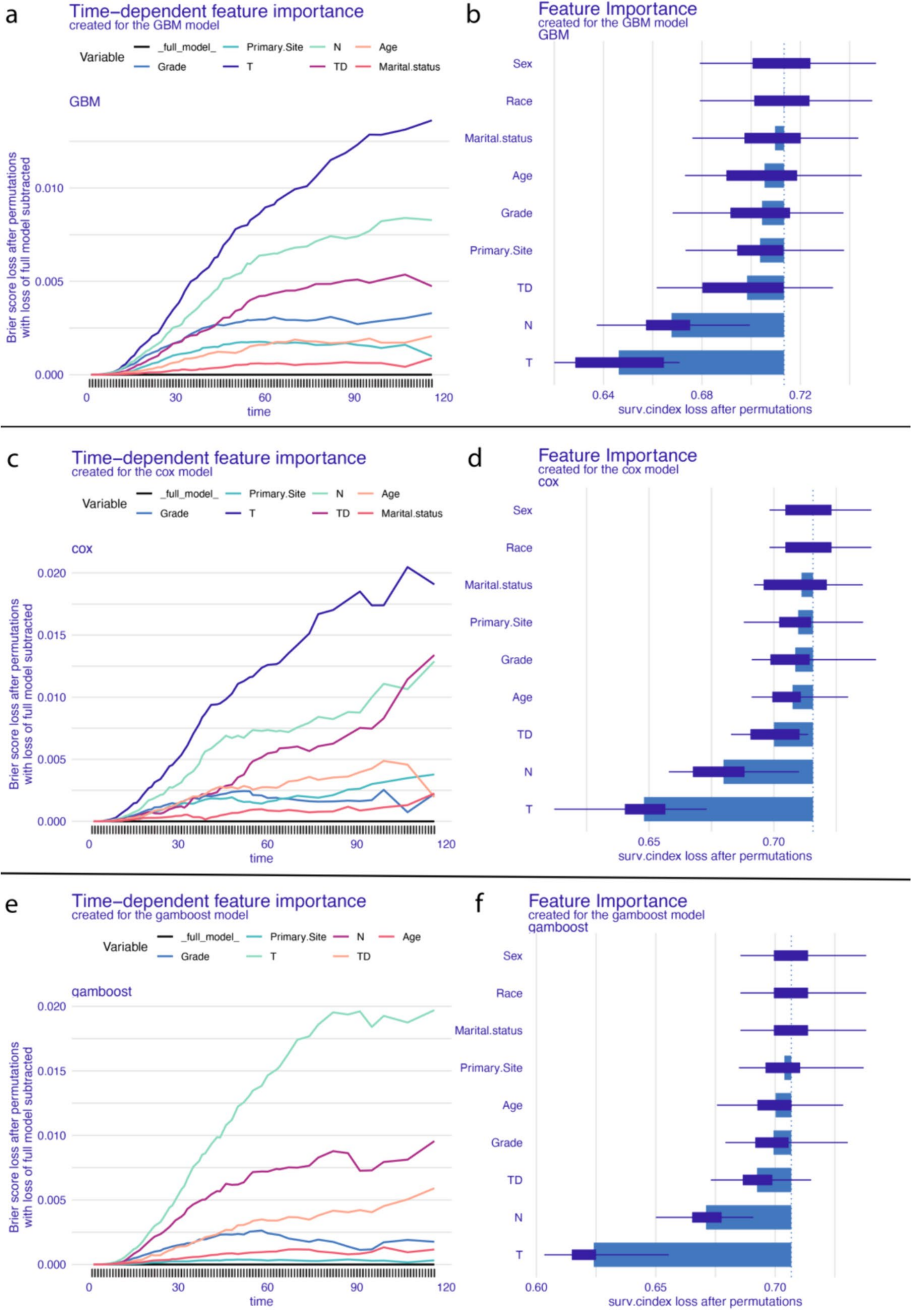
Time-varying feature importance in GBM (**a**), Cox (**c**), and GAMboost (**e**) models for colorectal cancer survival prediction; feature importance ranking for GBM (**b**), COX (**d**), and GAMboost (**f**) models predicting colorectal cancer survival

SHAP values demonstrated time-varying feature importance (Fig. 5). For GBM, T and N stage importance increased then decreased over time, while TD importance steadily rose. Grade and age remained relatively stable (Fig. 5a). Similar patterns were observed in the Cox and GAMBoost models (Fig. 5c and e), with TD significance progressively increasing. Overall, even though TD’s importance is not as significant as T or N in the early stages of survival, its importance gradually increases over time and may even surpass the N stage in some models.

In the feature ranking plot (Fig. 5), within the GBM model, the T stage, N stage, and TD are the top three most important features. Age, grade, and primary site are moderately important, while marital status, race, and sex appear to be less important for the model (Fig. 5b). Similar results can be observed in the Cox model (Fig. 5d). In the GAMboost model, T stage, N stage, and TD are the three most important features, followed by grade and age. Primary site, marital status, and other features have lower importance (Fig. 5f). This analysis demonstrates that, overall, TD is the third most important factor for the survival prediction of colon patients in all three models.

**Fig. 6:**
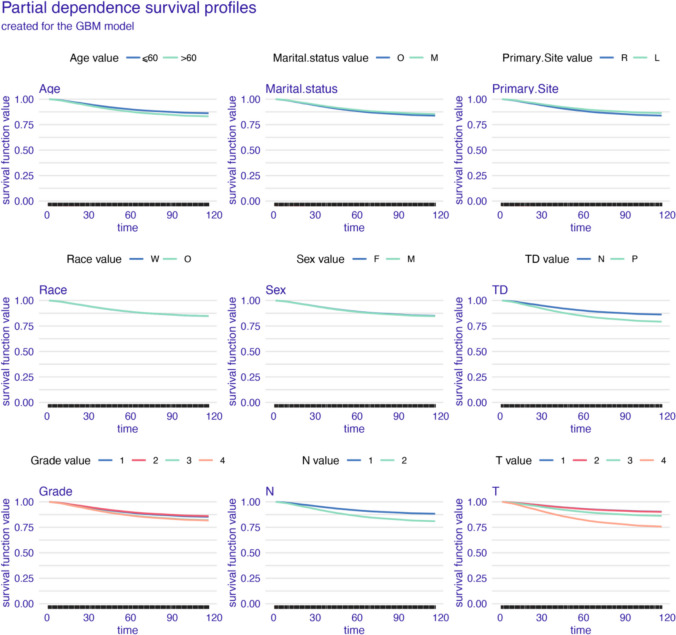
Partial dependence survival profiles for the GBM prognostic model

**Fig. 7:**
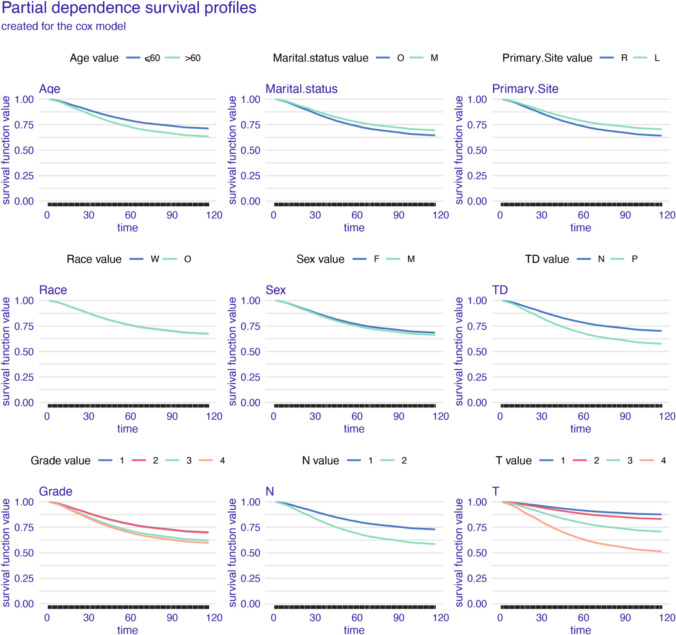
Partial dependence survival profiles for the COX prognostic model

**Fig. 8:**
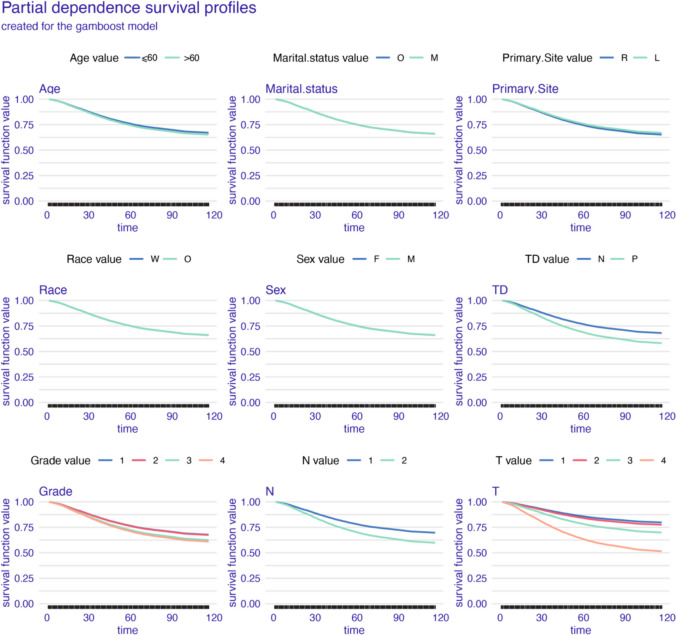
Partial dependence survival profiles for the GAMboost prognostic model

As shown in the partial dependence plots (Figs. 6, 7, and 8), all models show minimal survival impact of age, race, sex, and primary site. And higher histological grade emerges as a key differentiator of diminished prognosis. AJCC pT and pN stage stratification were also proved influential, alongside positive TD status being associated with poorer outcomes. However, distinctions arise in time-varying prognostic effects. The GBM model reveals dynamic relationships, with fluctuating TD significance and greater discrimination by AJCC pT and pN stage over time (Fig. 6). In contrast, the Cox and GAMBoost models demonstrate more static prognostic patterns for tumor factors (Figs. 7 and 8). In summary, while variable selection is aligned, GBM uniquely captures temporal shifts in the prognostic utility of pathological markers, specifically TD and histological grade.

## Disscusion

*Our study demonstrates TD as a highly significant prognostic indicator in NMLP-CA patients, evidenced by machine learning and Cox regression analysis*. Among evaluated models, GBM, Cox regression, and GAMBoost achieved optimal predictive performance based on metrics like c-index and calibration. The prognostic value of TD indicates potential limitations in the current AJCC TNM staging system for lymph node-positive patients. By integrating TD status alongside other key predictors, our machine learning models exhibit robust generalization ability for clinical application. They provide actionable insights on survival prediction to inform prognostic awareness and treatment decision-making.

In recent years, an increasing number of articles have indicated the association between cancer deposits and the prognosis of colorectal cancer [5, 6, 24, 25]. However, most current research primarily focuses on stage III colorectal cancer, with limited studies specifically investigating the role of TD in patients with NMLP-CA. Presently, scholars tend to consider TD merely as a substage component within the TNM staging system. The 7th edition of AJCC stipulates that “regardless of the number, size, or morphology of tumor deposits when they are present without accompanying regional lymph node metastasis, patients are classified as N1c stage[4]. However, when positive lymph nodes are present, only the count of positive lymph nodes is used for N staging.” The 8th edition extends the definition of N1c from the 7th edition [26]. The specific classification of tumor deposits in the TNM staging system remains a subject of debate. Lord et al. argue that the criteria for classifying TD under the N1c substage need adjustment, and they do not agree with the viewpoint that the presence of tumor deposits should be disregarded when positive lymph nodes are present [27]. Currently, some research suggests that combining the number of tumor deposits with the number of positive lymph nodes for N staging can enhance the accuracy of colon cancer staging [28]. Uneo et al. while comparing the impact of tumor deposits (TD) on T-stage categories and N-stage categories, found that the C-index confidence interval for T-staging was smaller than that for N-staging (0.6731–0.6760 vs. 0.6909–0.7167). Consequently, they proposed the inclusion of TD in N-staging [29]. Mirkin et al. conducted a retrospective analysis of stage III resectable colorectal cancer using the US National Cancer Database (2010–2012). They found that there was no significant difference in survival rates between patients in the T1-4 stage with TD ( +) and LN ( −) and those with LN ( +) and TD ( −). They suggested that tumor deposits should be considered lymph nodes and included in the N-staging [6]. Studies by Cohen et al. and Delattre et al. incorporated the number of TD into the lymph node count to create a new N-staging system. When comparing the prognosis of the newly restaged N2 with those initially classified as N2, both studies found similar outcomes [30, 31], and our study yielded similar results: we observed that the survival curves for patients with T1/2 and positive tumor deposits were similar to those of T3/4 and negative tumor deposits. Similarly, the curves for patients with N1 and positive tumor deposits resembled those of N2 and negative tumor deposits, suggesting that positive tumor deposits may elevate the T/N staging of patients. In addition to the existing N substage, Pricolo et al. proposed the staging of the “N2c” substage. They recommend categorizing patients with LN ( +) TD ( +) or LN ( −) TD ( +) with three or more tumor deposits as N2c stage, and those with LN ( −) TD ( +) and two or fewer tumor deposits as N1c stage [32]. Some scholars believe that considering TD ( +) as “N3” stage may better reflect its prognostic value in colorectal cancer [27]. Additionally, several studies have reported a high percentage of positive tumor deposits in node-negative (N0) cases [6, 33, 34]. This observation is of paramount importance and warrants further investigation. Several factors may contribute to this phenomenon, including the evolving definitions and diagnostic criteria of TDs, the inherent heterogeneity of colorectal cancer, the distinct biological characteristics of TDs compared to lymph node metastases, and advancements in pathological examination techniques [35–41].

Machine learning can enhance survival prediction efficiency, aiding disease prognosis and clinical decision-making [42, 43]. Compared to traditional analysis, machine learning better handles complex multidimensional data [44]. This study pioneered machine learning models using diverse prognostic features to predict the prognostic value of TD in NMLP-CA, demonstrating stable performance on test data and clinical utility. Additionally, we uniquely modeled time-varying feature importance with high accuracy, providing unprecedented insights into evolving predictor roles. Overall, this work highlights machine learning’s potential for advancing colon cancer outcomes via data-driven prognostic models.

This study has some limitations. The retrospective design may introduce biases, necessitating large-scale prospective validation of our models. We did not assess modern immunotherapies and targeted therapies, which represent prominent advances in colorectal cancer treatment [45–48]. Additionally, while race lacked prognostic significance in some models, our predominantly white cohort restricts generalizability across ethnicities. Moving forward, we aim to develop deep learning prognostic models by analyzing expanded, more diverse data. This could enhance risk stratification and personalized treatment for colon cancer patients.

In summary, there is currently a degree of controversy surrounding TD, from their definition to their clinical value, making it a hot topic in clinical research. Concerning clinical staging, the criteria for categorizing TD into the N1c substage may need adjustment, but there is no unified standard for including them in the T, N, or M substage. Most research results suggest that considering the combined count of tumor deposits and positive lymph nodes for N staging can improve the accuracy of colorectal cancer staging. While TD is associated with colorectal cancer prognosis, there is still no consensus on the cutoff values that define the number of tumor deposits affecting prognosis.

## Conclusion

In conclusion, this study identified tumor deposits (TD) as an independent prognostic factor for non-metastatic lymph node-positive colon adenocarcinoma (NMLP-CA), evidenced by Cox regression analysis. Machine learning models GBM and GAMBoost further demonstrated the significant prognostic value of TD for NMLP-CA patient survival. Given the potential influence of TD status on TNM staging, incorporation of TD as a parameter in future AJCC guidelines could improve prognostic performance. Overall, these findings underline the adverse impact of TD in NMLP-CA and support the expanded emphasis of TD in risk stratification for precision oncology.

## Data Availability

The original contributions presented in the study are included in the article/supplementary material. Further inquiries can be directed to the corresponding authors.

## Abbreviations

TD: Tumor Deposits
NMLP-CA: Non-metastatic Lymph Node Positive Colon Adenocarcinoma
SHAP: Shapley additive explanations
CSS: Cancer-specific survival
CRC: Colorectal cancer
AJCC: American Joint Committee on Cancer
LNM: Lymph node metastases
SEER: Surveillance, Epidemiology, and End Results
GLMboost: Generalized linear model boosting
Rfsrc: Random forestSRC
GAMboost: Generalized additive model boosting
DeepSurv: Deep learning survival analysis
OR: Odds ratios
CI: Confidence intervals

## References

[1] Bray F, Ferlay J, Soerjomataram I, Siegel RL, Torre LA, Jemal A (2018) Global cancer statistics 2018: GLOBOCAN estimates of incidence and mortality worldwide for 36 cancers in 185 countries. CA Cancer J Clin 68:394–424 30207593 10.3322/caac.21492

[2] Sung H, Ferlay J, Siegel RL, Laversanne M, Soerjomataram I, Jemal A, Bray F (2020) Global Cancer Statistics 2020: GLOBOCAN estimates of incidence and mortality worldwide for 36 cancers in 185 countries. CA Cancer J Clin 71(2021):209–249 10.3322/caac.21660 33538338

[3] Benson AB, Venook AP, Al-Hawary MM, Arain MA, Chen YJ, Ciombor KK, Cohen S, Cooper HS, Deming D, Farkas L, Garrido-Laguna I, Grem JL, Gunn A, Hecht JR, Hoffe S, Hubbard J, Hunt S, Johung KL, Kirilcuk N, Krishnamurthi S, Messersmith WA, Meyerhardt J, Miller ED, Mulcahy MF, Nurkin S, Overman MJ, Parikh A, Patel H, Pedersen K, Saltz L, Schneider C, Shibata D, Skibber JM, Sofocleous CT, Stoffel EM, Stotsky-Himelfarb E, Willett CG, Gregory KM, Gurski LA (2021) Colon cancer, Version 2.2021, NCCN clinical practice guidelines in oncology. J Natl Compr Canc Netw 19:329–359 33724754 10.6004/jnccn.2021.0012

[4] Cuccurullo V, Mansi L (2011) AJCC cancer staging handbook: from the AJCC cancer staging manual (7th edition). Eur J Nucl Med Mol Imaging 38:408–408

[5] Jin M, Roth R, Rock JB, Washington MK, Lehman A, Frankel WL (2015) The impact of tumor deposits on colonic adenocarcinoma AJCC TNM staging and outcome. Am J Surg Pathol 39:109–115 25229767 10.1097/PAS.0000000000000320 PMC4267920

[6] Mirkin KA, Kulaylat AS, Hollenbeak CS, Messaris E (2018) Prognostic significance of tumor deposits in stage III colon cancer. Ann Surg Oncol 25:3179–3184 30083832 10.1245/s10434-018-6661-9

[7] Liu Y, Zhang H, Wang Y, Wang C, Xiong H, Wang Y, Jing H, Jiang X, Hu H, Tang Q, Wang G (2022) How best to play the role of tumor deposits in stage III colon cancer? Front Oncol 12:860491 35296023 10.3389/fonc.2022.860491 PMC8918527

[8] Zheng P, Chen Q, Li J, Jin C, Kang L, Chen D (2020) Prognostic significance of tumor deposits in patients with stage III colon cancer: a nomogram study. J Surg Res 245:475–482 31446189 10.1016/j.jss.2019.07.099

[9] D’Ascenzo F, De Filippo O, Gallone G, Mittone G, Deriu MA, Iannaccone M, Ariza-Sole A, Liebetrau C, Manzano-Fernandez S, Quadri G, Kinnaird T, Campo G, Simao Henriques JP, Hughes JM, Dominguez-Rodriguez A, Aldinucci M, Morbiducci U, Patti G, Raposeiras-Roubin S, Abu-Assi E, De Ferrari GM, PS Group (2021) Machine learning-based prediction of adverse events following an acute coronary syndrome (PRAISE): a modelling study of pooled datasets. Lancet 397:199–207 33453782 10.1016/S0140-6736(20)32519-8

[10] Li W, Liu Y, Liu W, Tang ZR, Dong S, Li W, Zhang K, Xu C, Hu Z, Wang H, Lei Z, Liu Q, Guo C, Yin C (2022) Machine learning-based prediction of lymph node metastasis among osteosarcoma patients. Front Oncol 12:797103 35515104 10.3389/fonc.2022.797103 PMC9067126

[11] Weng SF, Reps J, Kai J, Garibaldi JM, Qureshi N (2017) Can machine-learning improve cardiovascular risk prediction using routine clinical data? PLoS ONE 12:e0174944 28376093 10.1371/journal.pone.0174944 PMC5380334

[12] Huang W, Xiao Y, Wang H, Chen G, Li K (2022) Identification of risk model based on glycolysis-related genes in the metastasis of osteosarcoma. Front Endocrinol (Lausanne) 13:1047433 36387908 10.3389/fendo.2022.1047433 PMC9646859

[13] Jiang J, Pan H, Li M, Qian B, Lin X, Fan S (2021) Predictive model for the 5-year survival status of osteosarcoma patients based on the SEER database and XGBoost algorithm. Sci Rep 11:5542 33692453 10.1038/s41598-021-85223-4 PMC7970935

[14] Li GQ, Wang YK, Zhou H, Jin LG, Wang CY, Albahde M, Wu Y, Li HY, Zhang WK, Li BH, Ye ZM (2021) Application of immune infiltration signature and machine learning model in the differential diagnosis and prognosis of bone-related malignancies. Front Cell Dev Biol 9:630355 33937231 10.3389/fcell.2021.630355 PMC8082117

[15] Zhu W, Xie L, Han J, Guo X (2020) The application of deep learning in cancer prognosis prediction. Cancers (Basel) 12(3):603. 10.3390/cancers12030603 10.3390/cancers12030603 PMC7139576 32150991

[16] Katzman JL, Shaham U, Cloninger A, Bates J, Jiang T, Kluger Y (2018) DeepSurv: personalized treatment recommender system using a Cox proportional hazards deep neural network. BMC Med Res Methodol 18:24 29482517 10.1186/s12874-018-0482-1 PMC5828433

[17] Wong-Chong N, Motl J, Hwang G, Nassif GJ Jr, Albert MR, Monson JRT, Lee L (2018) Impact of tumor deposits on oncologic outcomes in stage III colon cancer. Dis Colon Rectum 61:1043–1052 30086053 10.1097/DCR.0000000000001152

[18] Nagtegaal ID, Quirke P (2007) Colorectal tumour deposits in the mesorectum and pericolon; a critical review. Histopathology 51:141–149 17532768 10.1111/j.1365-2559.2007.02720.x

[19] Weiser MR (2018) AJCC 8th edition: colorectal cancer. Ann Surg Oncol 25:1454–1455 29616422 10.1245/s10434-018-6462-1

[20] Yabata E, Udagawa M, Okamoto H (2014) Effect of tumor deposits on overall survival in colorectal cancer patients with regional lymph node metastases. J Rural Med 9:20–26 25648159 10.2185/jrm.2880 PMC4310051

[21] Rock JB, Washington MK, Adsay NV, Greenson JK, Montgomery EA, Robert ME, Yantiss RK, Lehman AM, Frankel WL (2014) Debating deposits: an interobserver variability study of lymph nodes and pericolonic tumor deposits in colonic adenocarcinoma. Arch Pathol Lab Med 138:636–642 23902577 10.5858/arpa.2013-0166-OA PMC3935980

[22] Lundberg SM, Lee S-I (2017) A unified approach to interpreting model predictions. Adv Neural Inf Processing Syst 30. https://proceedings.neurips.cc/paper/2017/file/8a20a8621978632d76c43dfd28b67767-Paper.pdf

[23] Lundberg SM, Nair B, Vavilala MS, Horibe M, Eisses MJ, Adams T, Liston DE, Low DK, Newman SF, Kim J, Lee SI (2018) Explainable machine-learning predictions for the prevention of hypoxaemia during surgery. Nat Biomed Eng 2:749–760 31001455 10.1038/s41551-018-0304-0 PMC6467492

[24] Wang P, Song Q, Lu M, Xia Q, Wang Z, Zhao Q, Ma X (2022) Establishment and validation of a postoperative predictive model for patients with colorectal mucinous adenocarcinoma. World J Surg Oncol 20:330 36192778 10.1186/s12957-022-02791-z PMC9528152

[25] Yu C, Zhang Y (2020) Establishment of prognostic nomogram for elderly colorectal cancer patients: a SEER database analysis. BMC Gastroenterol 20:347 33081695 10.1186/s12876-020-01464-z PMC7576842

[26] Nicholls RJ, Zinicola R, Haboubi N (2019) Haboubi, Extramural spread of rectal cancer and the AJCC Cancer Staging Manual 8th edition, 2017. Ann Oncol 30:1394–1395 31046085 10.1093/annonc/mdz147

[27] Lord A, Brown G, Abulafi M, Bateman A, Frankel W, Goldin R, Gopal P, Kirsch R, Loughrey MB, Markl B, Moran B, Puppa G, Rasheed S, Shimada Y, Snaebjornsson P, Svrcek M, Washington K, West N, Wong N, Nagtegaal I (2021) Histopathological diagnosis of tumour deposits in colorectal cancer: a Delphi consensus study. Histopathology 79:168–175 33511676 10.1111/his.14344

[28] Brouwer NPM, Nagtegaal ID (2021) Tumor deposits improve staging in colon cancer: what are the next steps? Ann Oncol 32:1209–1211 34416364 10.1016/j.annonc.2021.08.1751

[29] Ueno H, Mochizuki H, Shirouzu K, Kusumi T, Yamada K, Ikegami M, Kawachi H, Kameoka S, Ohkura Y, Masaki T, Kushima R, Takahashi K, Ajioka Y, Hase K, Ochiai A, Wada R, Iwaya K, Nakamura T, Sugihara K (2012) Study group for tumor deposits without lymph node structure in colorectal cancer projected by the Japanese Society for cancer of the, rectum, multicenter study for optimal categorization of extramural tumor deposits for colorectal cancer staging. Ann Surg 255:739746 10.1097/SLA.0b013e31824b4839 22395093

[30] Cohen R, Shi Q, Meyers J, Jin Z, Svrcek M, Fuchs C, Couture F, Kuebler P, Ciombor KK, Bendell J, De Jesus-Acosta A, Kumar P, Lewis D, Tan B, Bertagnolli MM, Philip P, Blanke C, O’Reilly EM, Shields A, Meyerhardt JA (2021) Combining tumor deposits with the number of lymph node metastases to improve the prognostic accuracy in stage III colon cancer: a post hoc analysis of the CALGB/SWOG 80702 phase III study (Alliance)(☆). Ann Oncol 32:1267–1275 34293461 10.1016/j.annonc.2021.07.009 PMC8719434

[31] Delattre JF, Cohen R, Henriques J, Falcoz A, Emile JF, Fratte S, Chibaudel B, Dauba J, Dupuis O, Becouarn Y, Bibeau F, Taieb J, Louvet C, Vernerey D, Andre T, Svrcek M (2020) Prognostic value of tumor deposits for disease-free survival in patients with stage III colon cancer: a post hoc analysis of the IDEA France phase III trial (PRODIGE-GERCOR). J Clin Oncol 38:1702–1710 32167864 10.1200/JCO.19.01960

[32] Pricolo VE, Steingrimsson J, McDuffie TJ, McHale JM, McMillen B, Shparber M (2020) Tumor Deposits in stage III colon cancer: correlation with other histopathologic variables, prognostic value, and risk stratification-time to consider “N2c.” Am J Clin Oncol 43:133–138 31764018 10.1097/COC.0000000000000645 PMC7004443

[33] Agger E, Jorgren F, Joud A, Lydrup ML, Buchwald P (2023) Negative prognostic impact of tumor deposits in rectal cancer: a national study cohort. Ann Surg 278:e526–e533 36538637 10.1097/SLA.0000000000005755

[34] Jiang C, Shen Y, Xu C, Liu Y, Zhou H, Xu Q, Gu L (2024) Clinical and pathologic predictors of tumor deposits in colorectal cancer. J Gastrointest Cancer 55:182–187 38051392 10.1007/s12029-023-00988-3

[35] Amin MB, Greene FL, Edge SB, Compton CC, Gershenwald JE, Brookland RK, Meyer L, Gress DM, Byrd DR, Winchester DP (2017) The eighth edition AJCC cancer staging manual: continuing to build a bridge from a population-based to a more “personalized” approach to cancer staging. CA Cancer J Clin 67:93–99 28094848 10.3322/caac.21388

[36] Dienstmann R, Vermeulen L, Guinney J, Kopetz S, Tejpar S, Tabernero J (2017) Consensus molecular subtypes and the evolution of precision medicine in colorectal cancer. Nat Rev Cancer 17:79–92 28050011 10.1038/nrc.2016.126

[37] Guinney J, Dienstmann R, Wang X, de Reynies A, Schlicker A, Soneson C, Marisa L, Roepman P, Nyamundanda G, Angelino P, Bot BM, Morris JS, Simon IM, Gerster S, Fessler E, De Sousa EMF, Missiaglia E, Ramay H, Barras D, Homicsko K, Maru D, Manyam GC, Broom B, Boige V, Perez-Villamil B, Laderas T, Salazar R, Gray JW, Hanahan D, Tabernero J, Bernards R, Friend SH, Laurent-Puig P, Medema JP, Sadanandam A, Wessels L, Delorenzi M, Kopetz S, Vermeulen L, Tejpar S (2015) The consensus molecular subtypes of colorectal cancer. Nat Med 21:1350–1356 26457759 10.1038/nm.3967 PMC4636487

[38] Nagtegaal ID, Knijn N, Hugen N, Marshall HC, Sugihara K, Tot T, Ueno H, Quirke P (2017) Tumor deposits in colorectal cancer: improving the value of modern staging-a systematic review and meta-analysis. J Clin Oncol 35:1119–1127 28029327 10.1200/JCO.2016.68.9091

[39] Ueno H, Mochizuki H, Akagi Y, Kusumi T, Yamada K, Ikegami M, Kawachi H, Kameoka S, Ohkura Y, Masaki T, Kushima R, Takahashi K, Ajioka Y, Hase K, Ochiai A, Wada R, Iwaya K, Shimazaki H, Nakamura T, Sugihara K (2012) Optimal colorectal cancer staging criteria in TNM classification. J Clin Oncol 30:1519–1526 22430272 10.1200/JCO.2011.39.4692

[40] Ueno H, Mochizuki H, Hashiguchi Y, Ishiguro M, Miyoshi M, Kajiwara Y, Sato T, Shimazaki H, Hase K (2007) Extramural cancer deposits without nodal structure in colorectal cancer: optimal categorization for prognostic staging. Am J Clin Pathol 127:287–294 17210518 10.1309/903UT10VQ3LC7B8L

[41] Wang Y, Zhang J, Zhou M, Yang L, Wan J, Shen L, Liang L, Yao Y, Zhang H, Zhang Z (2019) Poor prognostic and staging value of tumor deposit in locally advanced rectal cancer with neoadjuvant chemoradiotherapy. Cancer Med 8:1508–1520 30790459 10.1002/cam4.2034 PMC6488131

[42] Miao R, Chen HH, Dang Q, Xia LY, Yang ZY, He MF, Hao ZF, Liang Y (2020) Beyond the limitation of targeted therapy: improve the application of targeted drugs combining genomic data with machine learning. Pharmacol Res 159:104932 32473309 10.1016/j.phrs.2020.104932

[43] Mosele F, Remon J, Mateo J, Westphalen CB, Barlesi F, Lolkema MP, Normanno N, Scarpa A, Robson M, Meric-Bernstam F, Wagle N, Stenzinger A, Bonastre J, Bayle A, Michiels S, Bieche I, Rouleau E, Jezdic S, Douillard JY, Reis-Filho JS, Dienstmann R, Andre F (2020) Recommendations for the use of next-generation sequencing (NGS) for patients with metastatic cancers: a report from the ESMO Precision Medicine Working Group. Ann Oncol 31:1491–1505 32853681 10.1016/j.annonc.2020.07.014

[44] Deo RC (2015) Machine learning in medicine. Circulation 132:1920–1930 26572668 10.1161/CIRCULATIONAHA.115.001593 PMC5831252

[45] Oshima K, Yamazaki K (2023) Immune checkpoint inhibitor therapy in neoadjuvant and adjuvant treatment for cancer: a paradigm shift in the treatment of resectable gastrointestinal cancer 3)A paradigm shift in the treatment of colorectal cancer. Int J Clin Oncol 28:1442–1450 37668816 10.1007/s10147-023-02387-x

[46] Pan Q, Fan X, Xie L, Wu D, Liu R, Gao W, Luo K, He B, Pu Y (2023) Nano-enabled colorectal cancer therapy. J Control Release 362:548–564 37683732 10.1016/j.jconrel.2023.09.014

[47] Xiao B, Yu J, Ding PR (2023) Nonoperative management of dMMR/MSI-H colorectal cancer following neoadjuvant immunotherapy: a narrative review. Clin Colon Rectal Surg 36:378–384 37795463 10.1055/s-0043-1767703 PMC10547541

[48] Zmuc J, Heil J, Herfarth C, Bechstein WO, Koch C, Trojan J, Schnitzbauer AA (2023) Chemotherapy and targeted therapy strategies in patients with unresectable or borderline resectable metastatic colorectal cancer: evidence for a lack of focus on resection rates. Ann Surg Oncol 30:7624–7632 37644249 10.1245/s10434-023-14049-3 PMC10562287

